# A qualitative exploration of roles and expectations of male partners from PMTCT services in rural Malawi

**DOI:** 10.1186/s12889-021-10640-z

**Published:** 2021-03-31

**Authors:** Beatrice M’baya Kansinjiro, Alinane Linda Nyondo-Mipando

**Affiliations:** 1 Department of Public Health, School of Public Health and Family Medicine, College of Medicine, Blantyre, Malawi; 2 Department of Health Systems and Policy, School of Public Health and Family Medicine, College of Medicine, Blantyre, Malawi

**Keywords:** Utilization, PMTCT, Adherence, Male involvement, Services, Roles

## Abstract

**Background:**

Prevention of mother-to-child transmission of HIV (PMTCT) is effective in curbing rates of HIV infection in children because its interventions reduce the rates of transmission during pregnancy, in labour, and in breastfeeding. Male involvement (MI) greatly influences uptake and adherence to PMTCT services. Lack of clarity on the roles and expectations of men in PMTCT is one of the main barriers to MI. The main aim of the study was to explore the roles and expectations of male partners from PMTCT services in Malawi.

**Methods:**

This was a descriptive qualitative study that involved men whose partners were either pregnant or breastfeeding a child, health care workers working in PMTCT services for over six months, and traditional leaders. We conducted 9 in-depth interviews and 12 key informant interviews from January to March 2018. All interviews were audio-recorded, transcribed, and translated. Thematic analysis was employed to analyze data.

**Results:**

The subjective and community norms and attitudes of men towards PMTCT provide the context in which male partners define the specific roles they render and the services they expect from PMTCT services. The roles of men in PMTCT service were contextualized in what is socially acceptable and normalized in the setting and include supportive roles expressed as accompanying the wife to attend; antenatal care services, Dry blood sample collection (DBS) when its due, keeping appointments when is due to take the ARVs, providing financial support; HIV prevention behavior change and decision-making roles. The desired services within PMTCT include health assessment such as checking their weight; blood pressure; blood sugar and promotion activities such as education sessions that are provided in a male-friendly manner that is in tandem with existing socio-cultural norms and attitudes of men towards such services.

**Conclusion:**

The roles of male partners in PMTCT services are underpinned by subjective norms and what is socially acceptable within a specific context. The services that men require from PMTCT services are influenced by their attitudes and beliefs towards PMTCT interventions. Services should be male-tailored provided in an atmosphere that allows and accepts male partners to exercise their roles in PMTCT services.

**Supplementary Information:**

The online version contains supplementary material available at 10.1186/s12889-021-10640-z.

## Background

Male Involvement (MI) is key to uptake and adherence of health services including PMTCT [1–4]. There are varied definitions for MI which include: the physical presence of a man in the antenatal or postnatal clinics, [5] and support that is remotely provided without accompaniment [1]. The support may include living positively with HIV infection either in HIV discordant or concordant couples; creation of a fast-track mechanism for women at the antenatal clinic because in most health facilities women who are accompanied by their partners at the antennal clinics receive preferential care by being served early than women who come alone; decision on the preferred method for family planning [1, 6]. Male involvement offers a platform for facilitated mutual disclosure of a couple’s HIV status and becomes socially appropriate for a woman to disclose her HIV-infected status due to the positive environment that men would provide [1, 6].

Although there are documented benefits that MI in PMTCT offers, such as, an increase in adherence and uptake of PMTCT services, HIV testing for both women and men; increase in the number of deliveries done at the hospital; prolonged breastfeeding, and male partner HIV counseling and testing [6–8], MI remains low [2, 8, 9] and ranges from 12.5 to 18.7% in Sub-Saharan Africa [10]. Previous studies in Sub-Saharan Africa have demonstrated that despite the efforts to encourage men to be involved in the PMTCT services only a few men respond positively by accompanying their female partners for antenatal services (ANC) and participating in PMTCT programmes [7].

Even with the documented strategies like engaging influential community leaders and community-based workers; creating male peer support groups; providing incentives such as checking blood pressure, deworming, height measurement, HIV and syphilis screening; and attending to couples earlier than women who come alone [11–13], the rates of MI have remained low. For instance, in Uganda implementation of the strategies only improved couple HIV testing rates from 12% in 2013 to 20% in 2015 [14].

Studies across Africa have illustrated that men fail to attend the PMTCT services because the services lack a male-specific-agenda [3, 7, 9]. One of the reasons for the suboptimal impact of MI as asserted by Theuring et al. (2009) is the lack of well-articulated roles of men within the ANC/PMTCT programmes which has resulted in men refraining from ANC/PMTCT clinics [3] leaving both health care workers and men unaware of what men ought to access when they attend PMTCT services with their partners. Given that there are no stipulated roles for men in PMTCT services, which create a gap in the services that are rendered when men accompany their wives to attend the PMTCT/ANC services; this study was conducted to explore the expectations of men in PMTCT services in a district in the southern part of Malawi. Specifically, we explored the perceived roles of men and specific services that men may access within PMTCT services. The findings from this study will feed into the PMTCT services as a way including men in the services. Additionally, the identified roles and services if incorporated in policy will serve as a benchmark for assessing MI in PMTCT initiatives in the country.

### Conceptual framework

This study was guided by the Theory of Reasoned Action which focuses on a person’s intention to behave in a specific way and provides an explanation of one’s change of behaviour and whether one will engage in a service or not [15]. In our study, the behaviour of interest was expressed as the roles of men and services they require in PMTCT services. The tenets of this theory include attitudes that are influenced by belief and evaluation of the belief; subjective norms which are influenced by a person’s perceptions on a particular belief and approval by others on the intended behaviour. The interface of attitudes and subjective norms shape the behavioral intentions which influence the actual or volitional behaviour that one shows [15]. In our study, we included an exploration of men’s understanding of PMTCT to assess their attitudes and social norms towards the service. We also assessed their belief in the PMTCT program to unravel the rationale behind involvement or non-involvement in PMTCT services. We further assessed their perceived roles in PMTCT which is influenced by how their significant others view their involvement in the program which is also embedded within the community norms. Cognisant that actual behaviour is driven by attitudes and subjective norms, we described the services that men require in the context of their attitudes towards the service and the congruence of the service with their subjective and community norms. This theory was selected because it facilitated the understanding of men’s attitude towards beliefs and further enabled the evaluation of how the beliefs and social norms affect the intention and the actual behaviors towards PMTCT services. It also guided the level at which the targeted services should be implemented such as individual, health facility, and the community to yield better involvement from men.

## Methods

### Study design

A descriptive qualitative study was conducted at two primary level health centres in the southern part of Malawi, from January to March 2018. We used In-depth interviews because they offer more time for a detailed and nuanced exploration of a phenomenon that facilitates understanding of complex issues and enables researchers to get an insight into the socio-cultural context of the participants [16, 17]. The interviews allowed us to gain insights from the people who are more knowledgeable about the topic to understand how certain things work [16].

### The setting of the study

Health facility A is located to the north while Health facility B is to the South-east of the District Hospital, both being Government-owned facilities and rural health centres. Health Centre A serves communities with a diverse educational background with several men employed in small companies, unlike Health Centre B where most men solely depend on working in farms and some informal employment. Male involvement at Health centre A is a voluntary act while at health centre B it is reinforced through the by-laws enacted by traditional leaders and the health care workers. Both sites offer the following services: outpatient services, low-risk ANC and deliveries; ART and PMTCT services; under-five clinics; drug dispensing, and have a referral system to the district hospital. At Health Centre A, initial booking for ANC is done every Monday while subsequent visits are done every Wednesday and Friday while at Health centre B, the initial visit is done every Tuesday, while subsequent visits are conducted on Wednesday and Thursday. There are 18 and 14 health care workers at Health centre A and Health centre B respectively.

### Sample size

We conducted a total of 21 in-depth interviews (IDI) among men, health care workers, and Village headmen. Baker argues that qualitative samples are usually small and that by the sixth interview, one should reach 70% saturation, and by the twelfth interview, the information reaches 92% saturation [18]. The definition of involvement included accompanying a partner for antenatal care services while non-involvement was defined as never accompanied by one’s partners for antenatal care services. Of the 9 men included in the study, 6 were involved in PMTCT and 3 men were not involved in PMTCT services. We also interviewed 8 health care workers and 4 village headmen.

### Selection of study participants

We drew a purposive sample because it allowed us to include participants with rich information on the issue that was being investigated [16]. Men who were involved in PMTCT were approached at antenatal and under-five clinics where they had accompanied their wives to access the PMTCT services. The three men that have never accompanied their partners to the hospital were approached from their respective homes. The PI approached men, guided by the eligibility criteria, on the day of their arrival at the clinic, early in the morning explained about the study, and asked them to come back for the interviews after receiving the services or the following day. Men who were not involved in PMTCT were approached in their respective homes with the assistance of community health care workers. The men were asked for their availability once they were interested in the study. We included men whose partners were either pregnant or breastfeeding babies of up to 24 months of age regardless of HIV serostatus, 18 years old and above, and with an expressed willingness to participate in the study.

Traditional leaders were approached in their respective homes with the assistance of community health care workers. Traditional leaders were only recruited into the study if they had been in their positions for at least six months regardless of gender, 18 years old and above, willing to participate in the study, and had a history of active involvement in Safe motherhood issues. The PI also booked an appointment on the first visit with the community worker, then would go to them for interviews on a date convenient for them. Health care workers were approached at their respective workplaces, with the assistance of the officers’ in-charge of each health centre, and were specifically selected because of their active involvement in providing PMTCT services. The PI booked an appointment with individual health care workers and was interviewed when off duty to avoid disrupting their duties. These included 2 Nurse Midwife technicians, 2 medical assistants, a PMTCT coordinator for the district, 2 Health Surveillance Assistants (HSAs), and 1 HIV Diagnostic Assistant (HDAs.) Health care workers were only recruited into the study if they had worked at their respective health facilities for not less than six months, 18 years old and above, and were willing to participate in the study. All participants were approached during their free time to avoid disrupting their work schedules.

### Data collection

We collected data using face-to-face interviews using topic-guides (see Additional File 1) that were translated into a local language. We used a digital audio recorder to capture all the interviews. The topic-guides were piloted at Health Centre C within the District. The broad questions that guided the interviews were:

- Would you please describe the roles of male partners in PMTCT services?
- Would you please describe the services that are required by men in PMTCT services?

After each broad question, we probed further to achieve a deeper understanding of the different opinions about the expectations of male partners in PMTCT services [16]. The Principal Investigator (PI) (BMK) conducted all the interviews. All interviews were audio-recorded and lasted for 30 to 45 min. All interviews were done in Chichewa which is the local language spoken in the area where we conducted the study.

The Principal Investigator was a Master of Public Health student who had training in qualitative research methods. There was no prior relationship between the researcher and the study participants and the PI introduced herself as a Master of Public Health student researching as part of her academic requirements. Before data collection, she informed the interviewees of the objectives of the study to promote the participants understanding of the subject. This was done to establish a good starting point for the interviews cognizant that at the time of the study there were no stipulated roles and services for men in the PMTCT programme in the facilities.

Data collection continued until saturation of ideas was reached, which was noted when no new ideas were expressed. Data saturation was reached when after preliminary analysis we realized that there was no more new information coming up and after which we conducted one interview to ascertain saturation of ideas. In the course of data collection, BMK and ALNM met and discussed the key outcomes to ascertain saturation of ideas. All the transcripts were simultaneously transcribed and translated verbatim into English. We used direct quotes from the transcribed and translated data from the audio-recorded interviews to clarify the responses from the participants and to ascertain the validity of the data [19]. At the end of each interview, the PI summarized the information provided by the participant to achieve the credibility of our findings [20]. We collected detailed field notes about the characteristics of the study sites, other interesting beliefs, and experiences of being in the field to enhance transferability. Since the research was part of a Master’s program, the Principal Investigator and the other researcher, listened and discussed the audios at different intervals of data collection to ensure that the study achieves its objectives. Only the researcher and participant attended the data collection session.

### Data analysis

We employed a manual thematic analysis [21]. One transcript was initially coded by the two authors to develop a coding framework. We manually color-coded the transcripts whereby similar codes were categorized and were later rearranged into themes. The researcher read the transcribed data multiple times to have a good grasp of the depth and breadth of the data while noting ideas of interest and, checking the transcripts back against the original audio recordings for accuracy [21]. Codes were inductively and deductively derived by identifying recurrent ideas as the data manifested and from the theory and objectives of the study respectively. Codes that had the same color from the manual coloring method employed were grouped to generate an overarching theme. Different codes were sorted into potential themes and the data extracts were put within the identified themes. This involved organizing all similarly coded data extracts into categories. The themes that were realized were charted in a thematic map (see Additional File 2) and were reviewed while paying attention to the emerging issues and any un-coded data was added at this stage bearing in mind that coding is a continual process. The process of coding was constantly discussed with the other Investigator to gain clarity and consensus [21]. We combined all themes that seemed to be related or alluding to the same idea and later named the themes influenced by the data under it.

## Results

### Socio-demographic characteristics of the participants

#### Demographic characteristics of male participants

The age of men ranged from 21 to 46 years; seven were married, seven were Christians, seven were unemployed, four had some primary education and two had secondary school education. None of the men we approached at a health facility refused participation while out of the eight men that we approached in their respective homes five of them refused participation and cited the fear of being followed up by health care workers to investigate their lack of participation in PMTCT services.

#### Demographic characteristics of health care workers

There were eight health care workers aged 29 to 65 years and five were females. Of the health care workers, four were nurses, one was a medical assistant, two were HIV diagnostic assistants, and one was the PMTCT coordinator of the district.

#### Demographic characteristics of village headmen

There were four village headmen, two from each of the study sites. They were all men with an age range of 49–70 years old. Two had attended primary school education and two never attended any formal education but were able to read and write.

#### Findings

Our findings are presented in the context of the Theory of Reasoned Action [15]. We summarized our findings under two major tenets of this theory because the interaction of the two is what shapes behaviour displayed by a person. The applicable tenets were subjective norms and attitudes, and our results show that these tenets are interlinked and related and influence each other. The theory assists in contextualizing the roles and services that men require from PMTCT services in a manner that is acceptable and desirable according to the setting where the services are rendered. This contextualization makes the services meaningful to the end-users.

#### Subjective norms that shape the roles of men in PMTCT

The participants’ beliefs and those approved by others were similar in some respects and also differed in other areas. The roles were relayed as activities that men play or are expected to play and were categorised in three areas namely, Supportive, HIV Prevention Behaviour, and Decision-making Roles

#### Supportive roles

The supportive roles expressed in the study varied and analysis showed that in some respects there was congruence from what the men said, what health care workers perceived and what the chiefs approved as culturally accepted and encouraged. Men are expected to financially provide for their families irrespective of a wife being pregnant or not and are expected to ensure that the family has food. When a wife or partner is pregnant then men are to provide for all necessary materials in preparation for the pregnancy and delivery.

*“As a man, I have a role to fund all the activities that may be needed, finding money for my wife to start antenatal care; for transportation; and buying necessary things for labour and delivery. Yes, that is my role.”* (Man 1, involved in PMTCT)

The health care workers also confirmed the role of a man to provide for his pregnant partner and this is shared with a pregnant woman as a list of items that a man ought to provide in preparation for the birth of the baby.

*“The man has to buy wrappers for the woman, to buy basins, soap for bathing and washing when the woman delivers. A man has that responsibility so he needs to find money to buy all that and other things like the food at home.”* (Health Worker, 2)

Health workers expect men to accompany their pregnant partners for antenatal services, delivery including under-five clinics. Health workers expect a man’s presence at every interface with the health system by the wife so that he is well informed.

*“The role of the husband is to accompany his wife to the PMTCT clinic. This makes the wife feel comfortable and relaxed which helps the baby to grow healthily.”* (Health worker 2)

Although men are expected at every interface a woman makes with the health system as expressed by health care workers, the men in the study found this concept to be new and contended that it needs wider implementation in the health services for it to be normalized as a man’s role which will facilitate acceptability of it among women, men and health care workers. Male involvement is not fully implemented despite being recommended in health facilities.

*“This issue just started sometime back, that men should be available when their wives are giving birth, I wish this issue was being implemented in our hospitals so that as men we should experience what our wives encounter. I just believe that if this happened we would have a common understanding with our wives and health care workers.”* (Man 1, involved in PMTCT)

In other cases, men are more motivated to accompany their partners when it is medically beneficial to them which cements that their action is primarily influenced by the belief in the output of the service. In instances where there is partner referral, men tend to show up to access the services and this was confirmed by both men and health care workers mainly because the medical condition normalizes their presence in a woman’s domain.

*“Honestly, I find it hard to accompany my wife to get tested for HIV, like today I came to receive medication because my wife tested positive for syphilis yesterday when she came for antenatal services.”* (Man 6, involved in PMTCT)

Village headmen also shared their experiences and stated that they are in support of men attending health care services with their partners.

*“We tell men to take their babies when they fall sick and most of them do that in this community. They are motivated because they do not wait for long hours, they are assisted early, so because of that men in this community play that role as well.”* (Village headman 4)

Implementation of supportive roles, particularly accompanying a woman to the health facility is deterred because, at the community level, it is not an acceptable norm. Men reported that they will be laughed at for escorting their partners because pregnancy and its related issues are traditionally a woman’s domain and if men are lingering there, they are deemed to be charmed, under a spell, or bewitched. There was a lack of consistency between what a community and individuals accept as a norm. The need to belong and behave as a community expects dissuades men from supporting their partners.

“*Do you think it is easy (to escort a wife to a health facility)? All men and women in this community laugh at a man who seems to be too busy with his wife and children, they mock you and segregate you to the point that you cannot have friends … .hmmm here you don’t dare, they speak when they have seen you* … .(Man 2, not involved in PMTCT)

Despite stating that men ought to accompany their partners, health care workers also acknowledged the lack of congruence between what is promoted by health care workers and what is socially accepted for men to do. When we probed on social underpinnings on male involvement, village headmen in both sites denied the presence of social norms in their communities that hinder men from accompanying their wives to ANC services.

*“There is no negative social influence that can stop a man from participating in the program. In this community we teach them that a man should love his wife whether at peace or not, we tell them to love their wives all the time and everyone has to abide by that’* (Village headman 2)

Seemingly a male partner may easily support his partner within the confines of their home and in more discrete tasks that will not be viewed against the norms in a community or that will not induce ridicule. Men and health workers agreed that male partners have the role of reminding their wives to attend antenatal care visits. However, the men who are not involved in PMTCT and the village headmen at both sites remained silent on this issue.

*“A husband has to remind the wife, right. When it is time to take drugs, she may be hesitant, he has to make sure that his wife is taking drugs.”* (Health Worker 5)

There was no agreement as far as household chores are concerned with Village headmen and men who were not involved in PMTCT emphasizing that such chores are for women, while some men who were involved stated that they participate in the chores.

*“In our community, all the household chores are considered to be for women, there let me not lie, for as long as you are married, if by any chance they see you washing, even your clothes, it is taken as a taboo, and the relatives of the woman do not allow that.”* (Village headman 1)

### HIV prevention behaviour roles

In the context of PMTCT, men are expected to take up HIV prevention strategies to ensure that their families are protected. Health care workers and men involved in PMTCT emphasized that it was a man who was to uphold faithfulness in a couple which slightly differed from what a man who was not involved in PMTCT stated

*“The role of the man is to make sure that he does not engage in promiscuous behaviours, he should not have extramarital affairs and he has to faithful to his wife to prevent from contracting the virus be it when the mother is not pregnant, is pregnant or breastfeeding.”* (Man3, involved in PMTCT).

However, men who are not involved in PMTCT viewed faithfulness as responsibility for both partners

“*We both have to be faithful, I mean a man and wife, we should trust each other and rely on each other. We should be open to each other to meet the needs of each other.”* (Man 2, not involved in non-PMTCT)

There was a discordance in the views expressed by men and health care workers’ overuse of condoms when a woman is pregnant. Health care workers emphatically specified initiation of condom use by men as a critical role for men to avoid infection especially when a couple is HIV infected, however, men viewed condoms as only necessary for family planning methods

“*So, the man has a responsibility to initiate the use of the condoms in the family when the family is HIV positive, be it during the time the wife is not pregnant, or is pregnant or is breastfeeding.”* (Health worker 8)

### Decision making roles for the family

Men are key in deciding on uptake of health services and there was a higher degree of congruence over this role and it resonated with what health care workers expect, with what men believe to be their primary role, and with what the community and cultural norms approve of them. Men as providers of their families viewed themselves as decision-makers for their families. Decision-making was discussed at different levels of PMTCT services including taking an HIV test. Men decide whether a couple and their children will undertake an HIV test or not and this was stated by all the various groups in the study. A village headman explained his assertions over it from the perspective of cultural beliefs that recognizes a man as the head of a household. He narrated as follows:

*“ [HIV] Testing should not wait for a woman to become pregnant, the role of a man, as the head of the family, is to decide on how often the couple should be tested to prevent the child from the virus.”* (Village headman 2)

Again, this decision is founded on the economic responsibility vested on a man as stated below:

“*We have to decide where our wives should attend ANC services because even though in Malawi these services are free, we still have to think that how will my wife reach there? What about during the rainy season how will she travel to reach there? So that she has to attend at the convenient possible hospital to prevent absenteeism*.” (Man 3, involved in PMTCT)

#### Attitudes towards male-specific services required in the PMTCT programme

The services suggested are classified under two broader themes and these are Health Assessment and Health Promotion Services. The participants further outlined how the services can be organized and delivered at both the health facility and community level.

##### Theme 1: health assessment services

The health assessment services men expected from a PMTCT service were Physical Assessment and Medical Consultation services. Male partners and health care workers at both sites suggested that men should have targeted health care services. Such services include checking blood pressure, body weight, and blood sugar.

*“If the health care workers are willing to help, I would love to have my blood pressure checked.”* (Man 2, involved in PMTCT)

Health care workers reported that men expect to be attended to and be given health advice as appropriate.

*“They expect to be asked if they are doing fine and given time to explain the problems that they may have. When men come to the antenatal clinic, they become our clients and we need to listen to their complaints and attend to them and refer if the need arises. We always have clinicians whom we work with, those should also be consulted.”* (Health worker 2)

However, some men who were involved and some who were not involved in PMTCT services had different opinions from other participants and emphasized that they do not require services that would benefit them directly. The choice of services could be stemming from their cultural beliefs on PMTCT being a woman’s domain. They insisted that they are just escorting their wives and that it is time-consuming for the healthcare workers to provide some services to the men.

*“We men are here just to escort our wives, so when you are just escorting you cannot request for anything. Antenatal is for pregnant women to should see how much the unborn baby weighs, therefore for us to be tested for no reason is impossible. Besides it is time-consuming for the health care workers.”* (Man 6, involved in PMTCT)

### Delivery of health assessment services

Participants suggested that the services should be delivered in a manner that is acceptable at either the health facility or community level and which will resonate with a positive attitude which as per this theory or reasoned action may result in using it. Men asserted that they expect the services and staff to be male-friendly. Their understanding of male-friendly facilities and services emphasized the organization of services like HIV testing, Blood pressure monitoring, and information.

#### Re-arrangement of clinic activities-targeted services

The health care workers and men from both sites stated that men would rather have the antenatal songs which are sung during each antenatal clinic session be omitted because they remain inconsistent with cultural norms of men singing and clapping hands. They recommended that health care workers should proceed with sharing the necessary information directly with the recipients. The economic responsibilities of a man may also influence this suggestion so that they are freed earlier to attend to other responsibilities.

*“You know what? At the ANC clinic, they sing songs and clap hands, I don’t like that. How do you expect me to sing songs in the presence of a large group of women, who are strangers for that matter?”* (Man 2, not involved in PMTCT)

However, the opinions of some male participants involved in PMTCT and village headmen were contrary to what was suggested as follows:

*“The hospitals are just ok, the services are also ok, there is nothing wrong with how they offer their services, as long as they receive the care they were supposed to, that is enough. Some men are just stubborn, they don’t need to be listened to.”* (Village headman 1)

#### Couple specific structural services

Health care workers and men who are involved in PMTCT advocated for a designated reception area for couples only because such services would be acceptable to men. This strategy will avert non-attendance at the services due to shyness stemming from services being offered in the presence of other women which is regarded as culturally inappropriate.

*“I feel shy when I sit together with strange women; I don’t become open. I would love if they can provide a space for couples only.”* (Man 2, involved in PMTCT)

#### Privacy and confidentiality in service provision

As a measure of achieving male friendly health services, especially with HIV services that men may access through PMTCT services, men and health care workers reported that the services have to be offered in rooms that maintain privacy which is largely influenced by the need to maintain cultural appropriateness of not being present in a woman’s domain. Men who are involved in PMTCT, health care workers, and some village chiefs at both sites also highlighted that male partners expect privacy because they feel relaxed in a closed environment

*“I expect to find a hospital that has rooms that provide privacy, and not only inside but also outside so that when entering into the room, people should not see you entering into the room where HIV testing is done. That is what I expect!”* (Man 1, involved in PMTCT)

Furthermore, participants suggested that the health care workers must be able to keep the information of people and services accessed confidential, to allow participants to be more open to them.

*“I think sometimes they (health workers) also need to be discreet, men should not hear from anywhere about the treatment that they have received at the hospital.”* (Health worker 2)

Safeguarding confidentiality becomes paramount in instances where health workers and men know one another as explained below:

*“Some of the health workers who conduct HIV tests are our colleagues, so we tend to wonder what would happen if we are diagnosed with the virus. You see unless they are confidential enough but otherwise we can be everyone’s talk.”* (Man 2, not involved in PMTCT)

#### Personalized and integrated services

Only men who are involved in PMTCT reiterated that men expect to be assisted by one health care worker who would provide all the necessary services without referrals from one health care worker to the other.

*“I expect to be assisted by one health care worker. Thus, when I arrive, he or she should educate me, counsel me and my wife, and do the check-up for my wife’s pregnancy. That should continue until when I come for the next visit, I should be booked the date that the healthcare worker who assisted me will be around.”* (Man 4, involved in PMTCT)

Additionally, health care workers and men further described the process of rendering services which to an extent highlights the role of men as decision-makers and household heads. They are likely transferring the same position and expect it to apply even in PMTCT services. This was expressed in the quotes below

*“They (men) want us to warmly welcome them, explain things about child preparation politely, they need to be helped fast so that they don’t have to wait for long hours at the clinic.”* (Health worker 6)

The health care workers and Village headmen affirmed that a receptive and respectful welcome would encourage more men to attend the PMTCT programme.

*“If we welcome them [men] nicely when they come, when they go home they will tell their friends and then they too will come. Do not judge the way they look. At times, we (health care workers) tend to judge saying they are too old, or maybe too young.”* (Health care worker 5)

At a community level, it was recommended that services should be integrated with sporting activities because they are an acceptable norm for men in most communities such that including health messages in them is desirable and will not be met with demeaning sentiments from others.

*“Through soccer, before the game starts there should be an announcement to talk about the MI in PMTCT issue. Encourage questions and tell them where they can access the services. I believe this would help to reach out to other men who may be ignorant or unwilling to participate.”* (Man 6 involved in PMTCT)

##### Theme 2: health promotional activities

The health promotion activities suggested were on educating and empowering men on their role and the services they can access from PMTCT services. Men suggested having education sessions concerning their health as part of the services.

*“Give us [men] health education concerning diseases such as malaria, STIs and how one can take care of oneself … because you may not know this from home but if you go to the hospital then you will know, and the health care providers should help with that.”* (Man 5, involved in PMTCT)

Men who are involved in PMTCT and health care workers services suggested that the information should be staggered in phases, unlike the current practice where participants are loaded with information on one visit.

“*I expect a healthy baby, being born without HIV. But our concern is that the information is given just once when we come for the first visit, the information is not repeated when we return and we wonder if we are doing the right things that the health care workers expect us to do, for us to have a child who is not HIV infected.”* (Man 2, involved in PMTCT)

### Delivery of health promotion services

The health care workers and men who are involved in PMTCT at both sites suggested sharing information using leaflets, brochures, or charts so that men could be reading when they are in the waiting area in the antenatal clinic as opposed to educating them.

*“The hospital can buy leaflets that have information about PMTCT, the roles that men are supposed to play and some benefits, just as it is with the male circumcision leaflet which gives a person the whole information about it and one does not need to ask about it”* (Man 5, involved in PMTCT)

Additionally, men and health care workers recommended the sharing of information on PMTCT services through television or radio that is located within a health facility. This approach was deemed inclusive because it would cater to men that cannot read on their own.

*“ If there is a radio or television, like at the district hospital in the children’s ward there is a radio or television, while they wait to be attended to, men could be watching or listening to that (information on PMTCT services) then they will be enticed. For those who can read then we can make some charts so they can read, as they wait to be assisted.”* (Health worker 2)

Giving health talks about the PMTCT services in areas where most men patronize, such as at Antiretroviral clinics and Outpatient Department is another way of delivering health promotional services. This approach was suggested by health care workers who noted that in most cases PMTCT education services are offered at the antenatal or under-five clinics where only a few men are in-attendance because men find such services culturally inappropriate.

*“In every opportunity that we have to meet men, at the hospital we should be able to give them the information. We can also find a way of spreading information about PMTCT, through OPD or ART. We tend to meet a lot of men in these areas at this hospital.”* (Health worker 7)

Village headmen and men involved in PMTCT services recommended the creation of peer support groups as a mechanism for peer-education to fellow men on PMTCT services in the villages. The participants stated that peer-education is effective in relaying information to others.

*“People love to learn from the people that they know, so if we agree to train some few men about PMTCT services so that they can be trained and train individuals … they will help the chief in advocating for this in the villages with other men. Then they will be helping the village headmen, I think this will be good if added.”* (Village headman 2)

Another form of sharing of information suggested was through collaboration with the community leaders such as traditional leaders, influential people such as religious leaders, family clans, and employers to facilitate sensitization in community gatherings with the village headmen and existing community healthcare workers. This approach was suggested because cultural beliefs regarding pregnancy, child care, and roles of men towards the care of pregnancy are the ones hindering men from participating in PMTCT services hence the need to work with opinion leaders and key people within a community to begin to change the norms and attitudes towards such services.

*“Some cultures like some religions, disregard these things. They don’t go to the hospital, it would be good if the message also reaches the church leaders, and they should understand it very well so that they can teach their people.”* (Village headman 3)

## Discussion

The findings of this study show that subjective and community norms and attitudes of men towards PMTCT provide the context in which male partners define the specific roles they render and the services they expect from PMTCT services. The roles of men in our study were contextualized in what is socially acceptable and normalized in the setting and include supportive roles expressed as accompanying wife to attend; antenatal care services, Dry blood sample collection (DBS), keeping appointments for resupply of ARVs, providing financial support; HIV prevention behaviour change and decision-making roles. The desired services within PMTCT include health assessment such as checking their weight; blood pressure; blood sugar and health promotion activities such as education sessions that are provided in a male-friendly manner that is in tandem with existing socio-cultural norms and attitudes of men towards such services.

### Roles of men in PMTCT services

The supportive roles of men in PMTCT services that were suggested in our study are similar to findings from earlier studies that reported that the main role of men was financial providers for their families [22–25]. This role is emphasized during couple antenatal education, and a male partner is asked to provide resources as part of a birth preparedness plan [26]. Our findings on the role of men as financial providers are contrary to what was reported earlier in Malawi where men reported that their willingness to participate in PMTCT services was impeded by their role to support their wives financially [1]. This means that there is a lack of clear description of the roles of men in the services [27] or there are other socio-cultural underpinnings that have not been considered in the existing description.

Other roles of men that were highlighted in an earlier study and not in ours include the physical presence of a man at the clinic where PMTCT services are delivered and attending to clinic appointments and all health-related issues [1, 28]. The observed difference in the roles of men arises from the way men perceive and describe their roles in different contexts [5]. The varying clarity on definitions and roles necessitates a deliberate effort to specify in the PMTCT guidelines on the roles of men to promote understanding across the stakeholders in PMTCT services while remaining in alignment with the norms and attitudes in a specific area.

Our findings on the role of men in caring for their partners from pregnancy, delivery, and post-delivery resonates with a South African study that regarded accompanying partners to the antenatal clinic as a form of spousal support [5]. Although men in Malawi accompany their spouses to the antenatal clinic, they usually wait outside the clinic rooms while their partner accesses services [28]. As it was reported earlier by Nkuoh et al. [24], the men in our study advocated for couples attendance to PMTCT services because that enhances their understanding and uptake of the health-related aspects during pregnancy and it remains congruent with the subjective norms and attitudes that shape a man’s interaction with a service. Additionally, our findings on rendering social support, remain consistent with another study that reported that by accompanying a wife, a man would encourage and support the woman during the stress and discomfort of pregnancy [29]. We suggest that men be included in all the health services activities that a woman undertakes to promote full participation and a way of normalizing the practice in the services [28].

Although the village headmen in our study stipulated that there are no social underpinnings in the communities that disturb MI, some men stated that men are discouraged from providing social support due to prevailing social-cultural norms. This was also expressed by the health care workers who bemoaned that what is advocated in the hospitals is incongruent with the social norms of where these people are coming from. These findings cement what a Ghanaian study reported that PMTCT and antenatal care services are regarded as a woman’s fair in their communities [24]. Likewise, Koo K et al. (2013) also claim that social beliefs reinforce the perception that men who accompany their wives to ANC clinics are weak or bewitched. We assert that issues of MI should begin in the communities where social norms are constructed so that they are incorporated and become the norm [30]. Despite the positive attitudes towards the roles of men expressed in this study, social factors are a hindrance to the execution of the roles. Social norms relegate maternal and child-care aspects to women which remains consistent with findings by Adelekan (2014) who reported that men were unwilling to participate in PMTCT services because it was socially inappropriate [31]. Although assistance with household chores was highlighted in our study as well as an earlier study [5], the division of roles according to gender, prohibits men from assisting a pregnant or a nursing mother, because of the prevailing norm that household chores are for women [5]. Going forward in developing guidelines for roles of men in PMTCT, it will require the involvement of custodians of influential leaders to ensure that the guidelines are socially appropriate.

The role of men in reminding a woman of clinic activities and HIV prevention services highlighted in our study is consistent with what was reported by earlier studies [13, 32]. We, therefore, argue that male involvement in providing health-related support falls within the pathway of achieving HIV viral load suppression among women and protecting children from contracting the virus and will impact positively on the UNAIDS 95: 95: 95 goal which Malawi adopted [33]. Furthermore, our findings that men should lead in the uptake of HIV prevention practices support findings from a study done by Larsson (2010) who reported that mistrust in marriage is due to lack of faithfulness between partners [22]. Focusing on HIV prevention as a role of male partners in PMTCT services would encourage the adoption of desirable behaviours among men [34, 35].

Men are key decision-makers in a family and society [7, 25, 36]. Specifically, in this study, men make decisions concerning attendance in PMTCT services and couple HIV testing, place and mode of labor and delivery, and infant feeding choices. Similarly, other studies have reported that the involvement of the father postnatally has led to a longer breastfeeding duration [2, 37]. Osoti et al. (2014) argued that when men make decisions on health-related care for the mother and baby, it yields positive outcomes such as giving birth under a skilled health worker, practicing exclusive breastfeeding, uptake of effective contraceptives, and infant immunizations [38].

Although men are expected at every interface a woman makes with the health system as expressed by health care workers, men in the study found this concept to be new. Similarly, even though village headmen agreed with men being available all the time, men contended that it needs wider implementation in the health services for it to be normalized as a man’s role which will facilitate acceptability of it among women, men, and health care workers. Male involvement is not fully implemented, because despite being recommended in health facilities, health care workers are in one part unequipped on what to do with the men, and on the other hand, the hospital infrastructure is not conducive for MI. this is in line with what Kalembo et al. (2013), found that lack of space in health facilities does not provide a conducive environment for MI as far as privacy and confidentiality are concerned [8]. We recommend the creation of male-friendly spaces within the existing facilities.

### Services men require in PMTCT services

The need for physical assessments and medical consultation services as suggested in this study is similar to what a Ugandan study referred to as a male health package [14] while in South Africa they were termed as free male health checkups [30]. The specific assessment reported in this study like; blood pressure, blood sugar, body weight, health education, and consultation services; differed from what was recommended in Uganda as part of the male health package. The male health package in Uganda included the provision of deworming services and ensuring that couples that present at the ANC are prioritized in accessing care [14]. In our study, the deworming services might not have been mentioned because of a lack of knowledge of the services that might benefit them which is compounded with the fact that the Malawian health system emphasizes deworming among children and antenatal women only [39].

The requested services by men may be assigned to other support staff trained with the required skills to avoid overburdening the midwives. WHO (2010) recommended task-shifting to overcome shortages of staff in hospitals [40]. The provision of such services will be in line with the Malawi Ministry of Health’s (MOH) vision statement which advocates for health for all regardless of gender, race, age, disability, and residing place [41].

Our recommendation on the creation of male-friendly services cements what was reported earlier in Malawi [12]. Other studies considered making health facilities male-friendly by reducing waiting times or introducing weekend or evening services for couples [10, 27, 42]. This study did not find similar sentiments and this may be due to the different settings, the previous study was done in urban areas while this study was done in rural areas where most men are not employed and may easily avail themselves during weekdays for clinic appointments.

Delineating a specific area for couples to access health service as reported in our study confirms what a Sub Saharan Africa systematic review [25] and other studies that revealed that organizing specific clinics for pregnant couples would be an effective way to increase male involvement in PMTCT activities because it achieves cultural appropriateness [10]. Similarly, it was noted that a lack of space to accommodate couples in consultation rooms was a barrier to MI because it made men feel uneasy when seated next to women they did not know [13, 25, 43]. Belato et al. (2017) advocated for separate waiting areas for men and women visiting maternal and child health clinics [44]. The current health systems of Malawi face a challenge of lack of adequate space to accommodate men in PMTCT service [27]. This suggests that there may be a need to engage other partners who may help in renovating the current health facilities and create enough space that would accommodate men in PMTCT services. This finding requires careful consideration because it may discriminate against women without partners or who are in unstable relationships [38]. Related to space was the need for privacy which has been reported in earlier studies [11, 27]. To achieve privacy, our study highlighted that HIV testing rooms should be behind other service rooms so that the areas should not be crowded and albeit safeguard privacy.

At the community level, services can be integrated with events that most men patronize like soccer. This approach ensures that men learn about their roles from health care workers in environments of comfort and aligned to cultural norms [11]. This approach would strengthen a community’s supportive attitudes towards PMTCT services while creating a conducive cultural environment that permits and expects male partners to be involved in PMTCT services [11]. To effectively deliver sessions on PMTCT services as men suggested in our study will require overcoming some barriers by bringing the required information to the people [45].

At the health facility level, the health promotion service messages can be delivered through audio-visual aid and provision of health education sessions in all areas men patronize such as outpatient department (OPD) or ART clinics. Similarly, earlier studies in Malawi stated that there is a need for health information sharing and male education about the importance of ANC and PMTCT [10, 27] and IEC has a key role in promoting MI in PMTCT [12, 46]. There is an association between exposure to media and uptake of HIV testing; more women and men who watched TV and women who read newspapers and listened to the radio daily took an HIV test than their counterparts who did not [46]. Information, education, and communication are vital in understanding issues and have proved to improve the attitudes of people on services [3, 47]. The provision of leaflets is common in the health system because they are already in use like in voluntary medical male circumcision (VMMC) services [48].

Optimal delivery of community services is influenced by prior stakeholders mapping and collaboration, integration within pre-existing services, and the creation of peer support groups. Earlier studies highlighted the provision of community services through collaboration with different stakeholders [10, 11, 25, 27, 49]. The stakeholders include church leaders, traditional leaders, and health care workers who can convey PMTCT messages in various forums [10, 25, 49]. It is believed that men are likely to change their views towards PMTCT when messages are shared at a church or a mosque which may increase participation in PMTCT services [11, 12, 24]. A multi-country study done in Sub-Saharan Africa reported that negotiating with community partners and working with leaders helped dismiss myths and fears around HIV and addressed challenges with stigma [49]. Similar approaches were described in the DRC and Côte d’Ivoire, where both community and religious leaders were lobbied to strengthen the capacity of those giving messages in the communities [11].

Equally, the study revealed that there is a need to collaborate with family members through their clan to promote male participation in PMTCT. Similar suggestions were reported by Besada et al. (2010) whereby the community members instilled powers to the people who were working in communities to sensitize the family on PMTCT with the potential of the message trickling toward their family members [11]. In Uganda, the collaboration of village elders and community leaders in the elimination of mother-to-child transmission (eMTCT) of HIV was expressed as fundamental in introducing a shift in the attitude of community members towards the role of men in ANC and eMTCT [14]. The creation of peer support groups in villages can facilitate male participation in PMTCT services as was previously reported in other studies [11, 22, 27]. The establishment of a male peer approach would be culturally appropriate for men to get information from their fellow men other than women [27]. This study did not find other support groups that have been advocated in PMTCT like expert clients are in general HIV care, [50] which is explained by the scope of this study whose reach was all men irrespective of their HIV status while expert clients are used in instances where one is HIV-infected.

### Strengths and limitations

The study has presented the opinions and perceptions of health care workers, men, and traditional leaders, which means that it provides a holistic approach to MI, however, this study excluded the voice of women who are an important party in MI in PMTCT. Although some men refused to participate in the study, we searched for more men to ensure that we have a purposive sample of what we studied. The sampling technique and study design employed does not allow for generalizations. Further research should follow an implementation method and be able to assess acceptability, feasibility, and fidelity of including the expectations of men in the PMTCT services.

## Conclusions

The roles of male partners in PMTCT services are underpinned by subjective norms and what is socially acceptable within a specific context. This study has revealed that there will be a great impact on MI in PMTCT related behaviours when PMTCT roles are socially accepted. Therefore, strengthening awareness of PMTCT services at both health facilities and in communities is more desirable. Additionally, stipulating the roles of men in PMTCT in necessary documents would guide the health care workers in knowledge and practice which will help them to meet the needs of male partners in PMTCT services. Further studies should focus on delivering a male-tailored package of interventions within PMTCT services.

## Supplementary Information

**Additional file 1.**

**Additional file 2.** Thematic Map for Roles and Services.

## Data Availability

The datasets used and analysed during this study are available from the corresponding author on reasonable request. The article was uploaded as a pre-print on: https://www.researchsquare.com/article/rs-5934/v1

## Abbreviations

ANC: Antenatal Care
ART: Antiretroviral Therapy
ARVs: Antiretroviral drugs
CDH: Chiradzulu District Hospital
CHAM: Christian Health Association of Malawi
CMA: Community Midwives Assistants
COM: College of Medicine
COMREC: College of Medicine Research Ethics Committee
DHO: District Health Officer
DIP: District Implementation Plan
GoM: Government of Malawi
HIV: Human Immunodeficiency Virus
HMIS: Health Management Information System
HAS: Health Surveillance Assistant
HTC: HIV Testing and Counseling
IDI: In-Depth Interviews
KII: Key Informant Interviews
MA: Medical Assistant
MCC: Maternal Child Care
MI: Male Involvement
PMTCT: Prevention of Mother to Child Transmission
TRA: Theory of Reasoned Action
