# Supplementary material for: A qualitative exploration of roles and expectations of male partners from PMTCT services in rural Malawi
Source: BMC Public Health. 2021 Mar 31;21:626. doi: 10.1186/s12889-021-10640-z (PMC8011175; doi:10.1186/s12889-021-10640-z)
Supplement: Supplementary file 1 — Additional file 1. [file 12889_2021_10640_MOESM1_ESM.docx]

## Interview guides for men

**Knowledge of HIV, HIV transmission, prevention and PMTCT**

1. Tell me, what do you know about Human Immune Virus (HIV)?

**Knowledge of PMTCT and Male Involvement in PMTCT services**

1. What do you understand PMTCT?
2. What does PMTCT involve?
3. Pleases explain to me what services are being offered in prevention of mother to child transmission of HIV?

Probe: How did you know about them?

1. What do you know about MI in PMTCT services?
2. Have you ever been involved in PMTCT services?

Probe on: Please explain how?

1. What is the importance of involving men in PMTCT services?

Ask why oneach importance

**Attitudes and beliefs of men towards PMTCT and MI in PMTCT services**

How do you view MI in PMTCT services?

How does your community view MI in PMTCT services?

**Perceived roles of men in PMTCT services**

We have talked about HIV, PMTCT, and Male involvement. Now let us talk about perceived roles of male partners in PMTCT services

1. What do you think are the PMTCT roles do men like you in this area currently play?

Ask why on each role

1. Probe on: before conceiving, during pregnancy, child birth and after-birth or under five care
2. Probe on roles played at home, health facility, (waiting area, during service provision)
3. Probe on how did you know about these roles
4. How often are you reminded about the roles, whereby whom?
5. Explain in details, what do you think are the key roles men are supposed to play inPMTCT at house hold, at community and facility level.
6. Please tell me, under what conditions are are you allowed to exercise about these roles.
7. Are there any other roles that men aught to play?

**Social norms**

1. What are the social norms that discourages /encourages you to to exercise your roles in PMTCT services?
2. Who makes decisions to seekmedical attention when your partner is pregnant? Why?

Probe on during labour and delivery, postnatal period, and underfive clinics.

**Expectations of men in pmtct services**

1. What about the hospital structure, what do you expectfromit?
2. Whatare your expectations your expectations on the clinic flow structure?
3. How about HIV testing services, how do you want themtobe offered?
4. Probe on as acouple? Or alone?
5. Whow about the healthcreworkers, what do you expect from them?
6. What are the actual services that concern your health? What do you expect from the health care workers?
7. What doyou expect fromyour community?
8. How about those women, who have come to access ANC services?

**Areas of Change**

Lastly let us talk about areas you would want to be improved, in provision of PMTCT services

1. Please explain clearly, if the Government of Malawi would want to revise the delivery of the PMTCT services, what do you think should be done differently to accommodate men in the programme?
2. What are any other issues that you want to talk about PMTCT and roles of men in the service? Its benefits?

End of the interview

## Interview guides for health care workers

**Type of Work**

Firstly I would like you to tell me more about your work:

1. What is your position or work title?
2. How long have you been working at this position?
3. What does your work at this health facility involve?

**Knowledge about male involvement and PMTCT**

1. What do you understand by PMTCT Please tell me,
2. What does OMTCT involve?
3. What do you understand by MI in PMTCT services?
4. Please explain, why is it important to involve men in PMTCT?
5. Please explain, how do you involve men in PMTCT services?

**Men’s understanding of PMTCT services**

1. Can you please explain in detail, the men’s undersrtanding of their involvement in PMTCT?
2. Explain the steps you follow at this facility in provision of PMTCT services?

**Health care workers perception on the roles of men in PMTCT services**

We have talked on how men understand PMTCT services; now let us discuss your perception towards the roles of male partners in PMTCT services.

1. Explain in detail, what you think are the roles of men in PMTCT services?
   1. ask why on each role
   2. Probe on PMTCT related roles at home
   3. Probe on PMTCT roles at the facility (waiting area, service provision)
   4. Probe on PMTCT roles before conception, during pregnancy, during labour and delivery, postnatal care and under five care
2. Now tell me, how did you know about these roles?
3. Explain in detail, what can you say about the adherence of men to the roles in PMTCT services?
4. As a health care worker, how do you encourage men to exercise and adhere to their roles in PMTCT services?

**Expectations of male partners**

1. What are the expectations of male partners at the health facility?
2. What are the expectations of male partners concerning the PMTCT clinic flow?
3. What are the expectations of male partners, concerning couple HIV testing?
4. What do men expect from the health care workers?
5. What do men expect from the healthcare workers concerning the health services directly provided to them?
6. What do men expect from their communitues?
7. How about women who have come to access the PMTCT services?

**Areas of Change**

Lastly let us talk about areas you would want to be improved, in provision of PMTCT services

1. If the Government of Malawi was revising the delivery of the PMTCT services, what do you think should be done differently to accommodate men in the programme?
2. What are any other issues that you want to talk about PMTCT and roles of men in the service?

**End of the interview**

Thank you very much for your participation and providing me with this information. Good day.

## Interview guides for traditional leaders

1. How long have you been a community leader for this community?

Probe on what sort of leadership?

**PMTCT and MI**

1. Understanding of PMTCT services
2. How men are involved in PMTCT services
3. How do you support that?

**Roles**

1. What are the roles men play in this community?
   1. Ask why on each role?
2. How do you facilitate the roles?

**Expectations**

1. What are the expectations of men?
   1. Ask on each expectation
2. How do you support the expectations?

**Services**

1. What services need to be included?
2. Ask why on each service?

**Areas of Change**

Lastly let us talk about areas you would want to be improved, in provision of PMTCT services

1. If the Government of Malawi was revising the delivery of the PMTCT services, what do you think should be done differently to accommodate men in the programme?
2. What are any other issues that you want to talk about PMTCT and roles of men in the service?

**End of the interview**

Thank you very much for your participation and providing me with this information. Good day.
