# Supplementary figures and images for: A qualitative exploration of roles and expectations of male partners from PMTCT services in rural Malawi
Source: BMC Public Health. 2021 Mar 31;21:626. doi: 10.1186/s12889-021-10640-z (PMC8011175; doi:10.1186/s12889-021-10640-z)

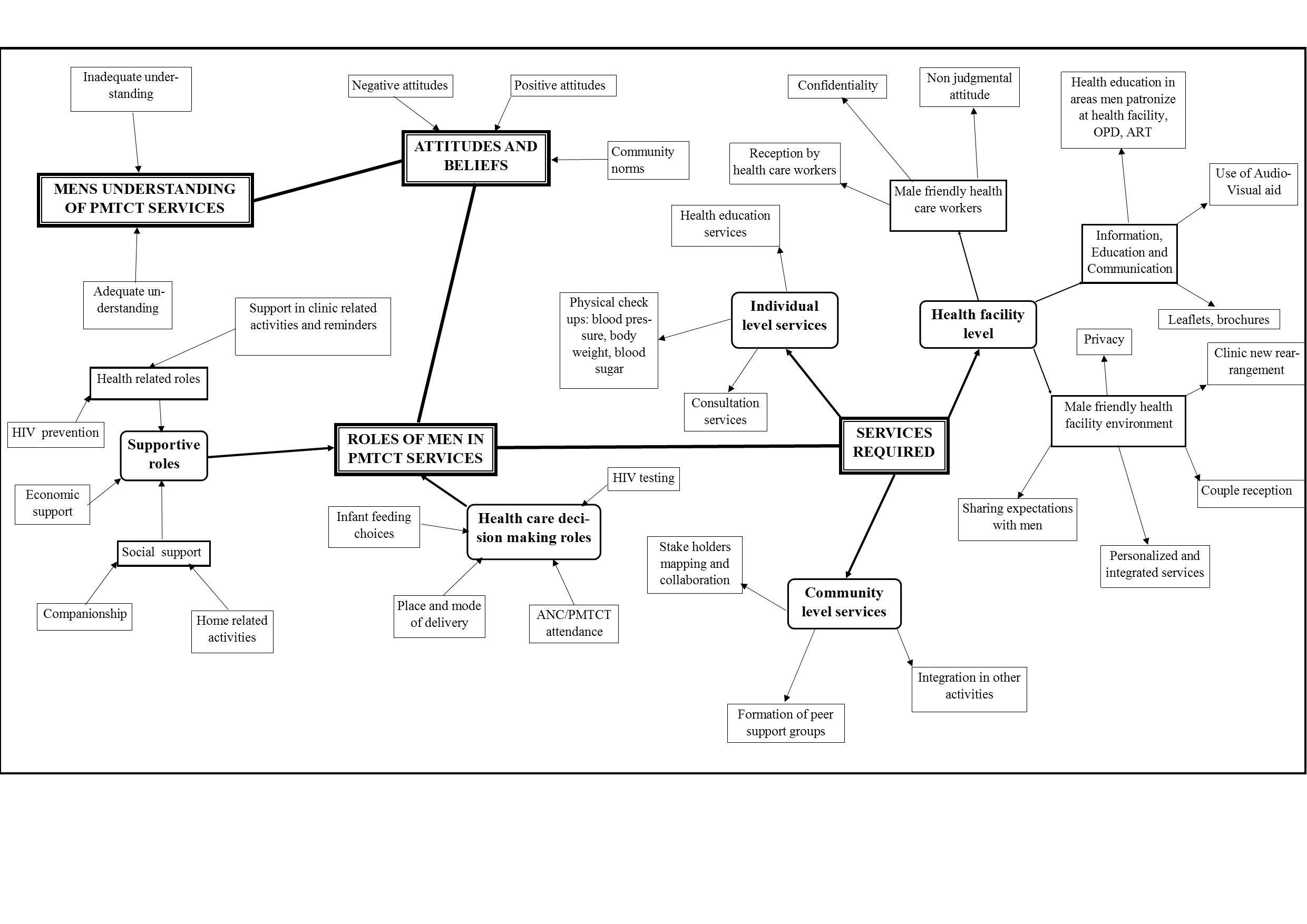


Thematic Map for Roles and Services

Supplement: Supplementary file 2 — Additional file 2. Thematic Map for Roles and Services. [file 12889_2021_10640_MOESM2_ESM.docx]
